# Activation of distinct inflammatory pathways in subgroups of LR-MDS

**DOI:** 10.1038/s41375-023-01949-2

**Published:** 2023-07-07

**Authors:** Marie Schneider, Clara Rolfs, Matthias Trumpp, Susann Winter, Luise Fischer, Mandy Richter, Victoria Menger, Kolja Nenoff, Nora Grieb, Klaus H. Metzeler, Anne Sophie Kubasch, Katja Sockel, Christian Thiede, Jincheng Wu, Janghee Woo, Andreas Brüderle, Lorenz C. Hofbauer, Jörg Lützner, Andreas Roth, Michael Cross, Uwe Platzbecker

**Affiliations:** 1 Department of Hematology, Cellular Therapy, Hemostaseology and Infectious Diseases, University Medical Center Leipzig, Leipzig, Germany; 2 Department of Medicine I, University Hospital Carl Gustav Carus, Dresden, Germany; 3 Novartis Institutes for BioMedical Research, Cambridge, MA USA; 4 Novartis Oncology, Basel, Switzerland; 5 UniversityCenter for Healthy Aging & Department of Medicine III, University Hospital Carl Gustav Carus, Dresden, Germany; 6 Department of Orthopedic Surgery, University Hospital Carl Gustav Carus, Dresden, Germany; 7 Department of Orthopedic Surgery, University Medical Center Leipzig, Leipzig, Germany

**Keywords:** Translational research, Myelodysplastic syndrome

## Abstract

Aberrant innate immune signaling has been identified as a potential key driver of the complex pathophysiology of myelodysplastic neoplasms (MDS). This study of a large, clinically and genetically well-characterized cohort of treatment-naïve MDS patients confirms intrinsic activation of inflammatory pathways in general mediated by caspase-1, interleukin (IL)-1β and IL-18 in low-risk (LR)-MDS bone marrow and reveals a previously unrecognized heterogeneity of inflammation between genetically defined LR-MDS subgroups. Principal component analysis resolved two LR-MDS phenotypes with low (cluster 1) and high (cluster 2) levels of *IL1B* gene expression, respectively. Cluster 1 contained 14/17 *SF3B1*-mutated cases, while cluster 2 contained 8/8 del(5q) cases. Targeted gene expression analysis of sorted cell populations showed that the majority of the inflammasome-related genes, including *IL1B*, were primarily expressed in the monocyte compartment, consistent with a dominant role in determining the inflammatory bone marrow environment. However, the highest levels of *IL18* expression were found in hematopoietic stem and progenitor cells (HSPCs). The colony forming activity of healthy donor HSPCs exposed to monocytes from LR-MDS was increased by the IL-1β-neutralizing antibody canakinumab. This work reveals distinct inflammatory profiles in LR-MDS that are of likely relevance to the personalization of emerging anti-inflammatory therapies.

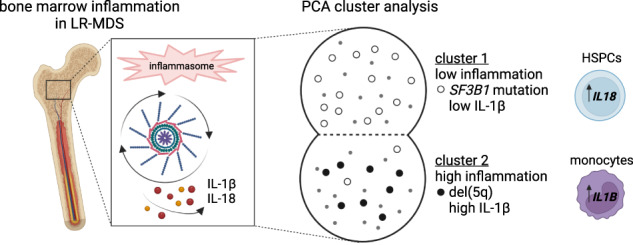

## Introduction

Myelodysplastic neoplasms (MDS) are a group of neoplastic clonal disorders of hematopoietic stem and progenitor cells (HSPCs) characterized by dysplasia and cytopenia [1, 2]. MDS can be preceded by clonal hematopoiesis of indeterminate potential (CHIP), in which clones marked by a pre-leukemic mutation are stably overrepresented in the blood in the absence of overt hematological disease [3]. Although MDS pathologies are heterogeneous, a growing body of evidence identifies dysregulated innate inflammation to be a common feature [4–6] that drives disease phenotype as well as disease progression *via* alterations occurring in both the hematopoietic and stromal compartments [7, 8].

Studies to date have focused on the activation of the NLRP3 (NOD-, LRR-, and pyrin-domain containing protein 3) inflammasome pathway in MDS and have shown how a self-perpetuating cycle of sterile inflammation can lead to progressive pyroptosis and dysfunction of the hematopoietic niche [9–11]. Specifically, elevated levels of pro-inflammatory cytokines, reactive oxygen species and alarmins such as S100A9 trigger NF-κB-driven expression of the inflammasome components NLRP3, PYCARD (pyrin domain and caspase recruitment domain) and caspase-1 together with pro-interleukin (IL)-1β and pro-IL-18. An activation signal such as extracellular ATP leads to the assembly of the multiprotein inflammasome complex, autoproteolytic activation of caspase-1, cleavage of pro-IL-1β and pro-IL-18, and release of the active cytokines.

The release of additional S100A9 from myeloid cells in response to IL-1β completes a positive feedback loop of sterile inflammation [12, 13] that has been proposed on the one hand to support the propagation of premalignant clones [14–17] and on the other hand to deplete hematopoietic progenitors by inducing pyroptotic cell death [18, 19].

An early study involving a limited number of patients has shown that both *IL1B* and *IL18* are expressed at high levels in low-risk (LR)-MDS [10], suggesting that sterile inflammation plays an important role in the establishment of LR-MDS and that suitably targeted anti-inflammatory therapies may have the potential to stall disease progression from LR- to high-risk (HR)-MDS and restore hematopoiesis. However, since MDS is a heterogeneous disease, the development of effective anti-inflammatory therapies suitable for a broad body of patients will require more detailed knowledge of the disease-specific inflammatory patterns, the contributions made by specific cell populations and the variation among LR-MDS patients [20]. A description of the heterogeneity of inflammation states in LR-MDS is likely to be a prerequisite for the stratification of anti-inflammatory treatments. Our objective was therefore to assess the diversity of bone marrow inflammation across a wide range of treatment-naïve LR-MDS patients compared to non-CHIP, CHIP and HR-MDS.

## Material and methods

### Patients and samples

Age-matched bone marrow samples from non-CHIP (*n* = 15) and CHIP (*n* = 12) healthy individuals (obtained from elective hip replacements; normal blood count; orthopedic patients within the BoHemE study) as well as treatment-naïve LR-MDS (*n* = 47) and HR-MDS (*n* = 14) patients were collected under written informed consent (according to the Declaration of Helsinki) as part of the MDS registry and the BoHemE Study (NCT02867085) at the University Hospitals in Dresden and Leipzig. All treatment-naïve MDS patient samples for which material, mutation status and clinical data were available were included in the study. MDS risk group was assigned according to the Revised International Prognostic Scoring System (IPSS-R) – LR-MDS: IPSS-R ≤ 3.5, HR-MDS: IPSS-R ≥ 4. Bone marrow mononuclear cells (BM-MNCs) were prepared by density gradient centrifugation. Molecular, cytogenetic and detailed clinical parameters were collected during routine diagnostics. Detailed cohort parameters are shown in Supplementary Table S1.

### Determination of inflammasome-related gene expression in bulk BM-MNCs by quantitative real-time PCR (qRT-PCR)

RNA was isolated from BM-MNCs using the AllPrep DNA/RNA Micro Kit (Qiagen, Hilden, Germany). Complementary DNA was generated using the Super Script™ IV VILO™ Master Mix (Invitrogen, Vilnius, Lithuania) and amplified using a customized TaqMan® Array (Thermo Fisher Scientific, Pleasanton, CA, USA) covering the inflammasome-related genes *S100A9*, *PYCARD*, *CASP1*, *IL1B*, *IL18*, *NLRP1*, *NLRP3*, *NLRC4*, *AIM2*, *CASP3*, *CASP4* and *CASP5*. PCR was carried out on a Quant Studio 5 device (Applied Biosystems) according to the manufacturer´s instructions. The mean of *HPRT1*, *GUSB* and *GAPDH* mRNAs were used for transcript normalization. Relative mRNA expression was calculated as log2 fold change relative to the mean of the non-CHIP samples. A cumulative gene expression score was calculated for each sample by summing all mRNA expression levels with the exception of *CASP5*, for which expression was undetectable for 15% of the LR-MDS samples.

### Cytokine analysis

Cytokine levels in bone marrow plasma were determined in duplicate using a customized 11-plex Luminex™ panel for IFN-α, IFN-γ, IL-1β, IL-1RA, IL-6, IL-8, IL-10, IL-17A, IL-18, CXCL10 and TNF-α (HCYTA-60K, Merck Millipore, Billerica, MA, USA) and single assays for HMGB1 and TGF-β (Merck Millipore, Billerica, MA, USA) on a FLEXMAP 3D™ system (Bio-Rad) according to the manufacturer´s instructions. Levels of S100A9 were determined in duplicate using a DuoSet ELISA (R&D Systems, Minneapolis, MN, USA) on a BioTek 800 TS plate reader according to the manufacturer´s instructions.

### Inflammasome-related gene expression analysis in sorted cell populations

Cryopreserved BM-MNCs of selected non-CHIP (*n* = 3) and CHIP (*n* = 3) as well as LR-MDS (*n* = 14) and HR-MDS (*n* = 5) cases (indicated in Supplementary Table S1) were thawed, stained and sorted into the following populations: CD45^+^,CD34^+^ (HSPCs); CD45^+^,CD14^+^,CD33^+^,HLA-DR^+^ (monocytes); CD45^+^,CD11b^+^,HLA-DR^-[stringent]^,CD14^+^,CD15^-/low^ (monocytic myeloid-derived suppressor cells, M-MDSCs); CD45^+^,CD11b^+^,HLA-DR^-[stringent]^, CD14^-^,CD15^+^ (polymorphonuclear myeloid-derived suppressor cells, PMN-MDSCs); CD45^+^,CD19^+^ (B lymphocytes); CD45^+^,CD3^+^ (T lymphocytes); CD45^–^ (control, non-hematopoietic and remnant erythroid cells). For detailed gating strategy and antibody specifications see Supplementary Fig. S1. Viable cells were sorted on a BD FACS Jazz directly into TRI Reagent® LS (Merck, St. Louis, MO, USA). RNA was isolated using the Direct-zol™ RNA MicroPrep Kit (Zymo Research, Irvine, CA, USA). Expression of the inflammasome-related genes *S100A9*, *NLRP3*, *PYCARD*, *CASP1*, *IL1B*, *IL18* and *NLRC4* was assessed by qRT-PCR using the GoTaq® 1-Step PCR kit (Promega, Madison, WI, USA) on a Quant Studio 5 device (Applied Biosystems) according to the manufacturer´s instructions. The primer sequences are shown in Supplementary Table S2. Transcript levels were normalized to *U6* mRNA. Reaction conditions were: 15 min at 50 °C for reverse transcription followed by 10 min at 95 °C and 40 cycles of amplification (10 sec at 95 °C, 30 sec at 61 °C, 30 sec at 72 °C). Relative mRNA expression was calculated as log2 fold change relative to the mean of the non-CHIP HSPCs.

### Colony-forming unit assay (CFU)

Cryopreserved BM-MNCs of *SF3B1*-mutated (*n* = 3) and del(5q) (*n* = 3) treatment-naïve LR-MDS patients (IPSS-R ≤ 3.5) were thawed and magnetically sorted for MDS-derived CD14^+^ monocytes (MACS, Miltenyi Biotec, Bergisch Gladbach, Germany). In parallel, CD34^+^ HSPCs were magnetically sorted from BM-MNCs of a healthy individual (MACS, Miltenyi Biotec, Bergisch Gladbach, Germany). MDS-derived monocytes were equilibrated at a density of 1 × 10^6^ cells/ml in StemSpan™ SFEM II + CC110 (STEMCELL Technologies, Vancouver, Canada) for 4 h prior to co-cultivation for 20 h with healthy HSPCs (ratio 10:1) in the same medium. A monoculture with HSPCs served as control. Where appropriate, cultures were supplemented with the IL-1β-neutralizing antibody canakinumab [100 µg/ml] or the NLRP3 inhibitor IFM-2384 [10 µM]. For the CFU assays 1.5 × 10^4^ cells (co-culture condition) or 1.5 × 10^3^ cells (HSPC monoculture as control) were plated in duplicate in the H4434 complete MethoCult™ medium (STEMCELL Technologies, Vancouver, Canada). After 10 days, erythroid, GM (granulocyte–macrophage) and GEMM (granulocyte–erythrocyte–macrophage–megakaryocyte) colonies were scored using an inverted light microscope.

### Statistical analysis

Statistical tests and graphical visualization were performed using GraphPad Prism version 8 (GraphPad Prism Software Inc.) unless otherwise stated. The spider plot was created using Microsoft Excel (Microsoft Corporation). Principal component analysis (PCA) and heat maps were created using R version 4.1.2. PCA on all mRNA expression values (exception of CASP5, for which expression was undetectable for 15% of the LR-MDS samples) was performed with the FactoMineR package version 2.4 and plotted using plotly version 4.10. Cluster determination was performed with NbClust version 3.0 with default settings [21]. The heat maps were generated using the ComplexHeatmap package v.2.2.0. The importance of the features in Fig. 4 was classified by Random Forest-based supervised learning approach with the Boruta package 7.0. [22]. The variable importance measure was calculated using a Random Forest approach and a *p* value of 0.01 was set as the confidence level. The importance of every single feature was confirmed by testing against permutated copies of the same dataset. The unimportant features were progressively eliminated. The Shapiro-Wilk test was used to test the results for normality. Comparisons of groups were performed using the Mann-Whitney test and the Kruskal-Wallis test followed by Dunn’s test to correct for multiple comparisons, the two-way ANOVA test followed by Tukey´s test to correct for multiple comparisons, the one-way ANOVA mixed-effects analysis with Geisser-Greenhouse correction followed by Tukey´s test to correct for multiple comparisons or the two-way ANOVA mixed-effects analysis followed by Sidak´s test to correct for multiple comparisons, as appropriate. Correlation analysis was performed by Spearman correlation. A *p* value  ≤  0.05 was considered statistically significant with the following significance levels: *p ≤ 0.05, **p ≤ 0.01, ***p ≤ 0.001, ****p ≤ 0.0001.

## Results

### Heterogeneous expression of inflammasome-related genes in CHIP and MDS

**Fig. 1:**
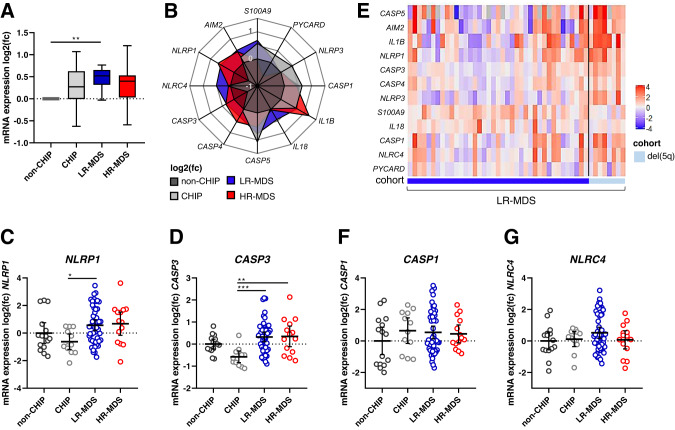
Inflammasome transcript profiling in healthy individuals and MDS patients. **A** Inflammasome-related gene expression is shown as pooled mRNA levels of all genes tested, expressed as mean log2 fold changes relative to the expression in non-CHIP BM-MNCs. Boxes show the distribution of mean changes for the 12 genes and whiskers show min. and max. values. **B** Spider plot of mean log2 fold changes (mean non-CHIP = 0) of individual mRNA expression in all cohorts. The mRNA expression values of **C** *NLRP1*, **D** *CASP3*, **F** *CASP1* and **G** *NLRC4* are plotted as log2 fold changes (mean non-CHIP = 0). Horizontal and vertical bars depict the mean and 95% confidence interval, respectively. **E** Heat map of log2 fold changes within the LR-MDS cohort with del(5q) cases grouped on the right. Cohorts: non-CHIP (*n* = 15), CHIP (*n* = 12), LR-MDS (*n* = 47) and HR-MDS (*n* = 14). Kruskal-Wallis test followed by Dunn’s test for multiple comparisons was applied to compare differences between all groups: *p ≤ 0.05, **p ≤ 0.01, ***p ≤ 0.001. CHIP clonal hematopoiesis of indeterminate potential, MDS myelodysplastic neoplasms, LR-MDS low-risk MDS, HR-MDS high-risk MDS.

Targeted expression analysis of inflammasome-related genes tested in LR-MDS BM-MNCs showed their overall pooled expression level to be 40% higher than that of non-CHIP controls (Fig. 1A), confirming the inflammatory state of LR-MDS bone marrow. Both CHIP and HR-MDS BM-MNCs expressed intermediate levels of inflammasome-related mRNAs, consistent on the one hand with a role for inflammation in the development of CHIP [23–25] and on the other hand with a decrease in inflammation when LR-MDS develops to HR-MDS [10, 26, 27].

The spider plot shown in Fig. 1B shows gene expression patterns for each disease state. The inflammasome-related genes most specifically associated with LR-MDS were *NLRC4* and *IL18*, while *IL1B* and *CASP4* expression were highest in HR-MDS. Somewhat surprisingly, we found expression of the genes most closely associated with the activity of the NLRP3 inflammasome (*NLRP3*, *PYCARD*, *S100A9*, *CASP1* and *IL1B*) to be increased in CHIP. In contrast, the CHIP samples expressed reduced levels of both *NLRP1* and *CASP3*, resulting in significant differences between CHIP and MDS states (Fig. 1C, D). These differences in gene expression suggest that it is not just the level of inflammasome related gene expression, but rather the topology of the network that differs between CHIP, LR-MDS and HR-MDS.

The heat map in Fig. 1E shows the wide range of expression levels of individual inflammasome-related genes within the LR-MDS group and suggests a link to genetically-defined subgroups, with relatively high gene expression in LR-MDS carrying del(5q).

Separate plots of mRNA levels of each gene (Fig. 1C, D, F, G and Supplementary Fig. S2) further illustrate the heterogeneity of gene expression levels within the LR-MDS cohort and reveal a dichotomous expression pattern for *CASP1* and *NLRC4* (Fig. 1F, G) that suggests the presence of at least 2 distinct inflammatory signatures within the LR-MDS cohort.

**Fig. 2:**
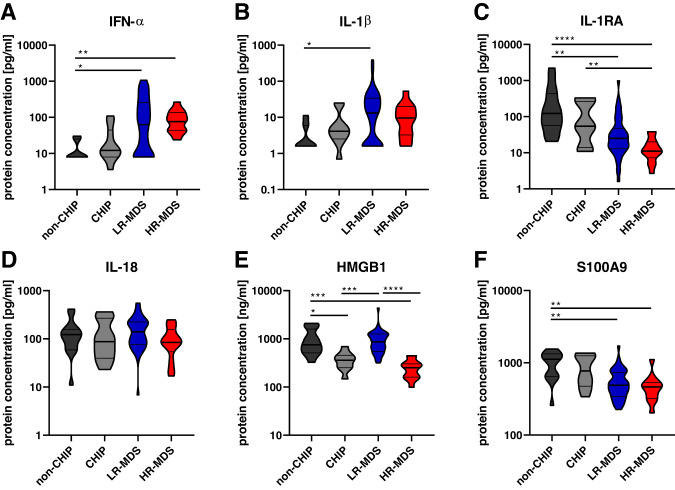
Protein measurement in bone marrow plasma samples. Violin plots of **A** IFN-α, **B** IL-1β, **C** IL-1RA, **D** IL-18, **E** HMGB1 and **F** S100A9 protein concentrations in bone marrow plasma samples. Bars depict the median (bold) and quartiles. Cohorts: non-CHIP (*n* = 15), CHIP (*n* = 12), LR-MDS (*n* = 47) and HR-MDS (*n* = 14). Kruskal-Wallis test followed by Dunn’s test for multiple comparisons was applied to compare differences between all groups: *p ≤ 0.05, **p ≤ 0.01, ***p ≤ 0.001, ****p ≤ 0.0001. CHIP clonal hematopoiesis of indeterminate potential, MDS myelodysplastic neoplasms, LR-MDS low-risk MDS, HR-MDS high-risk MDS, IFN-α interferon-α, IL interleukin, IL-1RA interleukin-1 receptor antagonist, HMGB1 high mobility group box 1, S100A9 S100 calcium-binding protein A9.

To complement the gene expression data, the levels of a range of secreted proteins associated with inflammation were tested in bone marrow plasma isolated from the same patient samples (Fig. 2 and Supplementary Fig. S3). Elevated bone marrow plasma levels of IFN-α (Fig. 2A) and IL-1β (Fig. 2B) further support the increased inflammation in CHIP and MDS. In contrast to *IL1B* mRNA, the bone marrow plasma IL-1β protein level tended to be lower in HR-MDS than in LR-MDS, possibly as a result of the progressive decline in the IL-1 receptor antagonist (IL-1RA) from CHIP through LR- to HR-MDS (Fig. 2C), in which IL-1β may therefore be more readily available to the receptor. IL-18 (Fig. 2D) varied only marginally between the disease states. There were clear and consistent differences in bone marrow plasma levels of the alarmin HMGB1 (Fig. 2E), with markedly reduced levels in both CHIP and HR-MDS, contrasting with near normal levels in LR-MDS. In contrast to a previous report [10], we found S100A9 protein levels in bone marrow plasma to be lower in the MDS samples than in the healthy controls (Fig. 2F).

### Inflammation states in LR-MDS correlate to disease genotype and clinical parameters

**Fig. 3:**
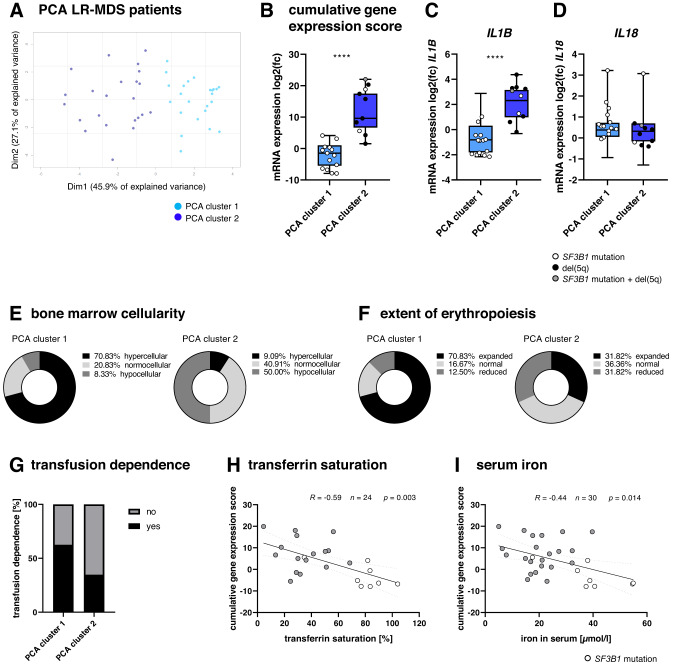
Distinct inflammatory subgroups within the LR-MDS cohort. **A** PCA clusters based on mRNA expression levels in bulk BM-MNCs of LR-MDS patients (*n* = 47): PCA cluster 1 (*n* = 24), PCA cluster 2 (*n* = 23). **B** Cumulative gene expression score per PCA cluster. Boxes show the median and whiskers show min. and max. values. **C** *IL1B* and **D** *IL18* mRNA expression values are plotted as log2 fold changes (mean non-CHIP = 0) per PCA cluster. Boxes show the median and whiskers show min. and max. values. Mann-Whitney test was applied to compare the difference between the PCA clusters: ****p ≤ 0.0001. **E** Bone marrow cellularity and **F** extent of erythropoiesis of LR-MDS PCA clusters are shown in pie charts. Extent of erythropoiesis is defined as GE-index: reduced ≥ 3.4, normal 1.5–3.3, expanded ≤ 1.5. **G** Distribution of transfusion dependence within LR-MDS PCA clusters. Correlation of cumulative gene expression score to (**H**) transferrin saturation and (**I**) serum iron concentration in LR-MDS patients. Spearman correlation with 95% confidence interval was applied and linear regression with 95% confidence bands is depicted. Dim dimension, MDS myelodysplastic neoplasms, LR-MDS low-risk MDS.

High inter-individual variation in the expression levels of a number of inflammasome-related genes and bone marrow plasma proteins within the LR-MDS cohort imply the existence of multiple inflammation phenotypes in LR-MDS that may correlate to the molecular and clinical characteristics of disease. In order to look further for functional relevance at the level of interacting networks, inflammasome-related gene expression data were subjected to a PCA. This led to the resolution of the LR-MDS samples into two groups of similar size (Fig. 3A) with *CASP1*, *PYCARD* and *NLRC4* contributing the most to both PCA dimensions (data not shown), consistent with the dichotomous expression patterns of *CASP1* and *NLRC4* noted above (Fig. 1F, G). The two groups were then compared in terms of overall inflammation by summing up the inflammasome-related gene expression levels for each sample and plotting the scores for each cluster. On this basis, the clusters represent low (PCA cluster 1, *n* = 24) and high (PCA cluster 2, *n* = 23) inflammation phenotypes (Fig. 3B). The samples in cluster 1 expressed low levels of *IL1B*, *NLRP3* (Fig. 3C, Supplementary Fig. S4A) and most other inflammation genes tested (Supplementary Fig. S4), but tend to express higher levels of *S100A9* (*p* = 0.062, Supplementary Fig. S4E). Also, although *IL1B* expression differed very significantly between the clusters (*p* < 0.0001, Fig. 3C), that of *IL18* did not (*p* = 0.788, Fig. 3D).

Fourteen of the 17 *SF3B1*-mutated samples located to cluster 1. In contrast, all 8 of the samples carrying del(5q) located to the high inflammation cluster 2, which also contained all 8 of the LR-MDS samples that had been found to lack any of the mutations covered by the standard myeloid panel (Supplementary Fig. S5). There was no significant difference in the incidence of *DNMT3A* or *TET2* mutation between the two clusters.

Comparison of clinical features revealed that patients in the low inflammation pattern PCA cluster 1 tended to have hypercellular bone marrow (Fig. 3E) and expanded erythropoiesis (Fig. 3F) as well as a stronger transfusion dependence (Fig. 3G), consistent with the predominance of *SF3B1*-mutated LR-MDS (14 of 24 patients) in this cluster. In contrast, patients in the high inflammation pattern PCA cluster 2 were mostly transfusion independent (Fig. 3G). A more detailed analysis of erythroid parameters was possible in a proportion of patients and confirmed that both transferrin saturation (Fig. 3H) and serum iron concentration (Fig. 3I) were inversely correlated to the cumulative inflammation gene expression score, being highest in *SF3B1*-mutated LR-MDS as previously reported [28].

### *SF3B1*-mutated and del(5q) LR-MDS samples showed distinct expression of IL-1β on mRNA and protein level

**Fig. 4:**
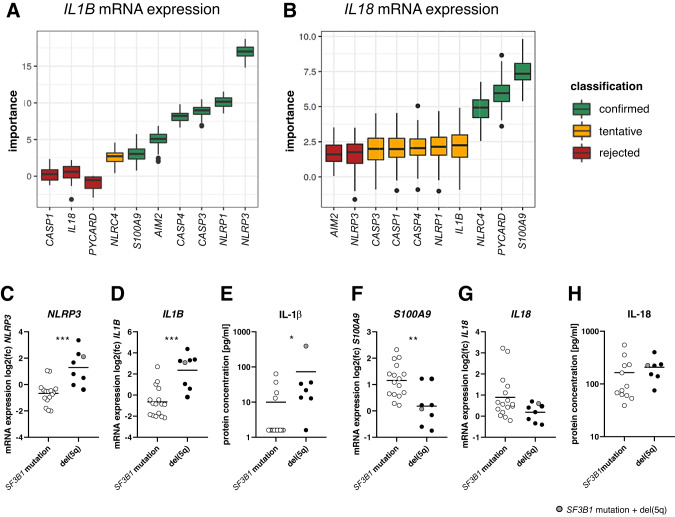
Gene expression associations in LR-MDS and comparison of individual gene expression levels between *SF3B1-*mutated and del(5q) cases. Associations between the expression of individual inflammasome-related genes and **A** *IL1B* and **B** *IL18* mRNA expression was classified by using the feature selection approach Boruta. Gene-specific importance to *IL1B* and *IL18* mRNA expression is displayed in box plots. Boxes show the median, whiskers show min. and max. values and dots represent outliers. **C** *NLRP3*, **D** *IL1B*, **F** *S100A9* and **G** *IL18* mRNA expression values in BM-MNCs as well as **E** IL-1β and **H** IL-18 protein concentrations in bone marrow plasma samples are plotted as log2 fold changes (mean non-CHIP = 0) per MDS genotype: *SF3B1* mutation vs. del(5q). Line shows the mean. Mann-Whitney test was applied to compare the difference between the MDS subtypes: *p ≤ 0.05, **p ≤ 0.01, ***p ≤ 0.001.

To examine in more detail the relationships between the main inflammasome effector cytokines IL-1β and IL-18, and other inflammasome components at the mRNA level, the feature selection algorithm Boruta was applied to the LR-MDS gene expression data to identify associations (Fig. 4A, B). This indicated that *IL1B* expression is most closely associated to that of *NLRP3*, followed by *NLRP1*, *CASP3* and *CASP4*, but is only weakly associated with *S100A9*. In contrast, *IL18* expression is associated most closely with that of *S100A9*, *PYCARD* and *NLRC4*. Figure 4C–H compare directly the gene expression of *NLRP3*, *IL1B*, *S100A9* and *IL18* as well as bone marrow plasma levels of IL-1β and IL-18 in LR-MDS carrying *SF3B1* mutation or del(5q), and reveal that *SF3B1*-mutated samples express significantly more *S100A9*, while del(5q) samples express significantly more *NLRP3* and *IL1B*, the latter also confirmed by IL-1β protein concentration.

### Monocytes, MDSCs and HSPCs contribute differentially to inflammatory gene expression

**Fig. 5:**
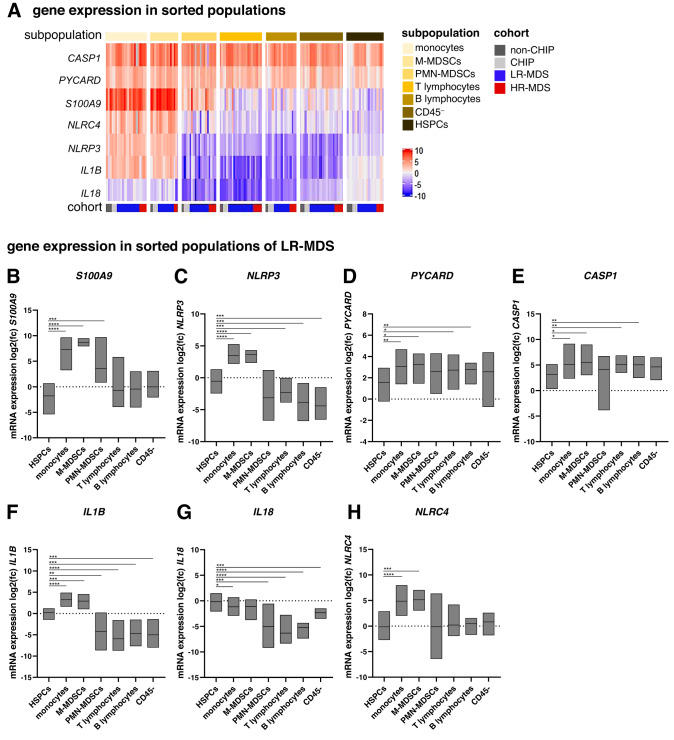
Inflammasome transcript profiling in sorted bone marrow populations. **A** Heat map of log2 fold changes (mean non-CHIP HSPCs = 0) of FACS-sorted bone marrow populations in all cohorts. mRNA expression values of inflammasome-related genes **B** *S100A9*, **C** *NLRP3*, **D** *PYCARD*, **E** *CASP1*, **F** *IL1B*, **G** *IL18* and **H** *NLRC4* in LR-MDS patients are plotted as log2 fold changes (mean non-CHIP HSPCs = 0). Floating bars show min. to max. values and line shows the mean. Mixed-effects analysis with Geisser-Greenhouse correction and Tukey’s multiple comparisons test was applied to compare differences between the sorted populations in LR-MDS patients. Significant differences relative to LR-MDS HSPCs are indicated here: *p ≤ 0.05, **p ≤ 0.01, ***p ≤ 0.001, ****p ≤ 0.0001. A comprehensive summary of significant differences between the various subpopulations is presented in Supplementary Fig. S6. Cohorts: non-CHIP (*n* = 3), CHIP (*n* = 3), LR-MDS (*n* = 14) and HR-MDS (*n* = 5). CHIP clonal hematopoiesis of indeterminate potential, MDS myelodysplastic neoplasms, LR-MDS low-risk MDS, HR-MDS high-risk MDS.

Given the differences in inflammasome-related gene expression between genetically distinct subgroups in LR-MDS, it was of interest to identify the cell types in which these genes are most active. To address this, HSPCs, monocytes, M- and PMN-MDSCs, B and T lymphocytes, and CD45^–^ cells (Supplementary Fig. S1) were FACS-sorted from the BM-MNCs of selected non-CHIP (*n* = 3), CHIP (*n* = 3), LR-MDS (*n* = 14) and HR-MDS (*n* = 5) samples (selected samples are indicated in Supplementary Table S1). LR-MDS samples were chosen to be representative of the two PCA clusters (*n* = 7 each). Given the limiting amounts of material available, qRT-PCR gene expression analysis was performed for a reduced panel of 7 inflammasome-related genes (*S100A9*, *NLRP3*, *PYCARD*, *CASP1*, *IL1B*, *IL18* and *NLRC4*) across all sorted populations from each of the 25 samples. The results are summarized in a heat map in Fig. 5A, which shows mRNA levels for each gene normalized to the mean level in HSPCs from the 3 non-CHIP donors. Figure 5B–H and Supplementary Fig. S6 present in more detail the expression data for the 14 LR-MDS samples. As expected, the monocyte and M-MDSC populations had the highest levels of expression of most of the inflammation genes tested. However, there were some exceptions. Firstly, the PMN-MDSCs expressed significant levels of *S100A9*. Secondly, *CASP1* and *PYCARD* were expressed across all populations and disease states at levels higher than in the non-CHIP HSPCs to which they were normalized. Both *CASP1* and *PYCARD* thus appear to become activated in HSPCs during the emergence of CHIP, consistent with the high level of expression in CHIP BM-MNCs shown above (Fig. 1 and Supplementary Fig. S2). Thirdly, the highest levels of *IL18* expression were found in HSPCs, which also expressed moderate levels of *IL1B*. Single-cell RNA-seq data from healthy donors confirmed *IL18* to be highly expressed in normal hematopoietic stem cells, while *IL1B* is more expressed in progenitor cells and differentiated myeloid cells, including granulocytes and monocytes. *NLRP3* expression is more restricted to myeloid cells, with modest expression in HSPCs (Supplementary Fig. S7) [29].

### The CFU activity of HSPCs exposed to MDS-derived monocytes in vitro is increased by selective inhibitors of inflammation

**Fig. 6:**
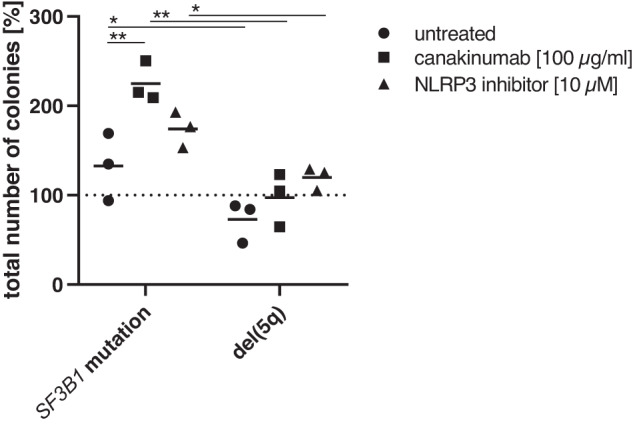
Colony-forming capacity under in vitro anti-inflammatory treatment. CFU assays of healthy donor HSPCs co-cultured with monocytes from *SF3B1*-mutated (*n* = 3) and del(5q) (*n* = 3) LR-MDS patients under in vitro anti-inflammatory treatment with canakinumab [100 µg/ml] and the NLRP3 inhibitor IFM-2384 [10 µM]. Total number of CFU colonies normalized to HSPC monoculture set as 100% (dashed line). Two-way ANOVA test followed by Tukey´s test for multiple comparisons was applied to compare differences between the treatment conditions within the same MDS disease type, while mixed-effects analysis followed by Sidak´s test for multiple comparisons was applied to compare the difference between MDS disease types: *p ≤ 0.05, **p ≤ 0.01.

The differential expression of inflammasome-related genes in LR-MDS is potentially relevant to the stratification of anti-inflammatory intervention strategies currently under clinical investigation. As a preliminary ex vivo test of response to anti-inflammatory drugs, we exposed healthy donor HSPCs to monocytes from *SF3B1*-mutated (*n* = 3) or del(5q) (*n* = 3) MDS bone marrow in the presence or absence of the IL-1β-neutralizing antibody canakinumab and the NLRP3 inhibitor IFM-2384. Following co-culture, the hematopoietic potential of the HSPCs was tested in colony assays (Fig. 6). Exposure of HSPCs to monocytes from del(5q) LR-MDS led to a decrease in subsequent colony formation compared to monocytes from *SF3B1*-mutated LR-MDS bone marrow, which may mirror the hypocellularity in the PCA cluster 2 (Fig. 3E). The differences between *SF3B1*-mutated and del(5q) derived monocytes were significant, suggesting that the higher overall inflammatory gene expression in del(5q) LR-MDS (Fig. 1E) results in a more detrimental effect on normal HSPCs. The inclusion of either the IL-1β-neutralizing antibody canakinumab or the NLRP3 inhibitor IFM-2384 in co-cultures resulted in increased numbers of colonies, reaching significance in the case of canakinumab treatment of co-cultures with monocytes from *SF3B1*-mutated MDS bone marrow, which showed significantly lower levels of *IL1B* gene expression (Supplementary Fig. S8). The overall increase in CFU activity was accompanied by a tendency to produce more erythroid cells at the expense of myeloid lineage outputs (Supplementary Fig. S9).

## Discussion

This study on a diverse cohort of LR-MDS patients confirms the intrinsic activation of inflammatory pathways in LR-MDS bone marrow [10] and identifies a number of previously unknown features of potential relevance to therapeutic stratification.

First, we found that the expression of genes closely associated with the NLRP3 inflammasome, including *NLRP3* itself, *IL1B*, *PYCARD*, *CASP1*, and *S100A9*, is already increased in CHIP. The analysis of sorted bone marrow cells revealed one of the most distinctive features of CHIP, an increased expression of both *PYCARD* and *CASP1* within the CD34^+^ population of HSPCs. The increased expression of inflammasome genes in CHIP is consistent with the association of CHIP with systemic inflammation that appears to contribute to the pathogenesis of cardiovascular and other diseases associated with advancing age [30–32].

While CHIP bone marrow shows signs of increased *NLRP3* expression, overall inflammasome-related gene expression was clearly highest in LR-MDS. Most importantly, our analysis of a relatively large number of patients revealed significant heterogeneity in the patterns of inflammation gene expression in LR-MDS. A dichotomous expression pattern of both *CASP1* and *NLRC4* implied the existence of at least two distinct inflammation phenotypes, which were confirmed by a PCA. The characteristics of PCA cluster 2 resemble those previously described for LR-MDS [10], with generally high levels of expression of inflammasome-related genes including *NLRP3* and *IL1B*. In contrast, PCA cluster 1 was characterized by a clear trend towards higher levels of *S100A9* gene expression but a lower level of inflammation gene expression overall.

Importantly, the two PCA clusters identified by distinct patterns of inflammasome-related gene expression also resolve distinct genetic entities in LR-MDS. All 8 of the del(5q) samples tested were in cluster 2, while 14 of 17 *SF3B1*-mutated cases were located in cluster 1. Our results therefore suggest that the *NLRP3*-*IL1B*-type of inflammation previously described for LR-MDS in fact applies to only half of all cases. These include all cases with the del(5q) genotype that is already recognized to be highly inflammatory [33, 34] and to progress readily to HR-MDS [35–37]. Indeed, the HR-MDS samples analysed in our study had high levels of *IL1B*, most closely resembling LR-MDS cluster 2.

Of note, all of the 8 LR-MDS samples lacking a common myeloid panel mutation also located to the high inflammation PCA cluster 2, while those carrying mutations other than *SF3B1* were evenly distributed. This implies the existence of further subgroups of inflammation phenotypes that remain to be characterized in more extensive studies.

The analysis of gene expression in sorted bone marrow cell populations confirmed that *IL1B* is most highly expressed in monocytes and M-MDSCs coincident with *NLRP3* that is responsible for cytokine activation and release. In contrast, we found *IL18* to be expressed predominantly in HSPCs, consistent with a previous report of increased *IL18* expression in ring sideroblasts in *SF3B1*-mutated MDS [38]. It is unclear which of the inflammasome proteins are most likely to contribute to the activation and release of IL-18 from HSPCs. Across all LR-MDS samples, the expression of *IL18* associated most closely with that of the alarmin *S100A9*, *NLRC4* and *PYCARD*. Although NLRC4 has previously been implicated in IL-18 activation in autoinflammatory disease [39–43], we found it to be expressed in the LR-MDS HSPCs at only very low levels. However, the HSPCs do express *PYCARD*, raising the possibility that PYCARD may play a role in the activation and release of IL-18 in cluster 1-type LR-MDS. It will clearly be important to trace the intracellular and intercellular interactions mediating inflammation in LR-MDS at the single-cell level in order to fully resolve the network complexity and diversity of disease states.

Finally, our functional co-culture test showed that anti-inflammatory agents indeed help to maintain HSPC function ex vivo in the presence of LR-MDS bone marrow monocytes. Specifically, inclusion of the IL-1β-neutralizing antibody canakinumab during co-culture of HSPCs with bone marrow monocytes from *SF3B1*-mutated LR-MDS significantly increased the subsequent colony forming activity of the HSPCs. These data also indicate that inhibition of the IL-1β pathway can improve erythropoiesis at the expense of myeloid output, as suggested in a biomarker study from the CANTOS trial [44]. The effects were less marked using the NLRP3 inhibitor IFM-2384 and in the case of co-culture with monocytes derived from del(5q) LR-MDS. Our unexpected observation that canakinumab appears to have a stronger effect on those cells that express lower levels of IL-1β suggests that IL-1β is probably a critical component of inflammation throughout LR-MDS but may be more difficult to inactivate in those that express high levels. While this highly simplified two-component culture system cannot recapitulate the complex inflammatory situation in vivo, the results do indicate a differential response of *SF3B1*-mutated and del(5q) monocytes to IL-1β inhibition. The recently activated CANFIRE trial (NCT05237713), in which LR-MDS patients are treated with canakinumab, will provide an opportunity to investigate such associations in more detail and in a clinical setting.

In conclusion, this work describes a diversity of inflammation states in LR-MDS bone marrow that is associated with distinct disease entities and is likely to be relevant to the stratification of emerging anti-inflammatory treatments.

## Supplementary information

Supplementary Data

## Data Availability

Original data are available upon request from the corresponding author.
