# Supplementary material for: Activation of distinct inflammatory pathways in subgroups of LR-MDS
Source: Leukemia. 2023 Jul 7;37(8):1709–18. doi: 10.1038/s41375-023-01949-2 (PMC10400420; doi:10.1038/s41375-023-01949-2)
Supplement: Supplementary file 1 — Supplementary Data [file 41375_2023_1949_MOESM1_ESM.pdf]

Supplementary Table S1. Detailed cohort parameters.

cohort	PCA cluster	WHO classification (2016)	sex	age	anemia	IPSS-R score	trans-fusions	mutation (VAF in %)	cytogenetics	RS
**non-CHIP**			M	74	no		no	none	NA	
**non-CHIP**			F	71	no		no	none	NA	
**non-CHIP**			F	79	no		no	none	NA	
non-CHIP			F	77	no		no	none	NA	
non-CHIP			F	64	no		no	none	NA	
non-CHIP			F	69	no		no	none	NA	
non-CHIP			F	63	no		no	none	NA	
non-CHIP			F	68	NA		no	none	NA	
non-CHIP			F	79	no		no	none	NA	
non-CHIP			F	68	no		no	none	NA	
non-CHIP			M	64	no		no	none	NA	
non-CHIP			F	67	no		no	none	NA	
non-CHIP			F	77	no		no	none	NA	
non-CHIP			F	60	no		no	none	NA	
non-CHIP			F	67	no		no	none	NA	
non-CHIP	median age [years] = 69.8 ; ratio F/M [%] = 86.7 / 13.3									
**CHIP**			F	77	no		no	DNMT3A (2.6/2.2/1.2)	NA	
**CHIP**			F	73	no		no	DNMT3A (5.8)	NA	
**CHIP**			F	64	no		no	BCOR (1.2), DNMT3A (3.2), TET2 (2.5), ZRSR2 (31)	NA	
CHIP			M	83	no		no	PPM1D (1.8), TET2 (3.4/1.5)	NA	
CHIP			M	76	no		no	DNMT3A (1.2)	NA	
CHIP			F	69	no		no	DNMT3A (3.4)	NA	
CHIP			F	85	no		no	TET2 (3.2/5)	NA	
CHIP			M	80	no		no	PPM1D (1.1), TET2 (2.1/3.3)	NA	
CHIP			F	72	no		no	DNMT3A (6.5)	NA	
CHIP			M	69	no		no	DNMT3A (1.2/2), IDH2 (7.4)	NA	

cohort	PCA cluster	WHO classification (2016)	sex	age	anemia	IPSS-R score	trans-fusions	mutation (VAF in %)	cytogenetics	RS
CHIP			F	79	no		no	TET2 (2.2)	NA	
CHIP			M	85	no		no	KRAS (22), TET2 (3)	NA	
CHIP	median age [years] = 76 ; ratio F/M [%] = 58.3 / 41.7									
**LR-MDS**	1	MDS-RS-MLD	F	88	yes	3	yes	SF3B1 (NA), TET2 (NA)	normal	yes
**LR-MDS**	1	MDS-RS-MLD	M	78	yes	2	yes	ATRX (100), SF3B1 (39.8), TP53 (52.8)	normal	yes
**LR-MDS**	1	MDS-RS-MLD	F	84	yes	3	yes	CUX1 (benign, 44.9), SF3B1 (46.4), TET2 (50.7/43.7)	normal	yes
**LR-MDS**	1	MDS-MLD	M	79	yes	3	yes	DNMT3A (8.2), SRSF2 (5.8)	normal	no
**LR-MDS**	1	MDS-RS-MLD	M	74	yes	3.5	yes	MPL (12.8), SRSF2 (42.7)	+19	yes
**LR-MDS**	1	MDS-MLD	M	69	yes	2	yes	ASXL1 (27.8), TET2 (88.4), ZRSR2 (93.6)	normal	no
**LR-MDS**	1	MDS-RS-MLD	M	79	yes	2.5	yes	DNMT3A (45), SF3B1 (44.3)	normal	yes
LR-MDS	1	MDS-RS-MLD	F	73	yes	2.5	yes	DNMT3A (38), SF3B1 (45), TET2 (6/44/25)	normal	yes
LR-MDS	1	MDS-RS-SLD	F	27	yes	3	yes	ZRSR2 (43)	normal	yes
LR-MDS	1	MDS EB1	M	50	no	3	no	IDH1 (39), PHF6 (16), RUNX1 (48)	-Y	no
LR-MDS	1	MDS-RS-SLD	F	74	yes	3	yes	SF3B1 (47)	normal	yes
LR-MDS	1	MDS-RS-MLD	M	67	yes	2.5	yes	SF3B1 (87)	normal	yes
LR-MDS	1	MDS-RS-MLD	M	61	yes	3	yes	SF3B1 (46)	normal	yes
LR-MDS	1	MDS-RS-MLD	M	74	yes	3.5	yes	SF3B1 (47.2)	normal	yes
LR-MDS	1	MDS-MLD	M	76	yes	2	no	PHF6 (11), TET2 (20)	normal	no
LR-MDS	1	MDS/MPN-RS-T	M	81	yes	2	no	JAK2 (17), SF3B1 (40), TET2 (23)	normal	yes
LR-MDS	1	MDS-RS-MLD	F	73	yes	1	no	SF3B1 (42.4)	normal	yes
LR-MDS	1	MDS-RS-MLD	M	68	yes	3.5	no	DNMT3A (44.9), SF3B1 (45.1), TET2 (47.6/17.2/4.5)	normal	yes
LR-MDS	1	MDS-MLD	M	49	yes	2.5	no	TET2 (21.1), ZRSR2 (81.5)	normal	no
LR-MDS	1	MDS-EB1	M	70	yes	3.5	yes	ASXL1 (26.5), STAG2 (80.9)	normal	no
LR-MDS	1	MDS-RS-MLD	M	82	yes	3.5	yes	DNMT3A (42), SF3B1 (38.5)	normal	yes
LR-MDS	1	MDS-RS-MLD	M	76	yes	3	no	CUX1 (51.2), DNMT3A (46.4), SF3B1 (46.3), TET2 (21.6), WT1 (46.8)	normal	yes
LR-MDS	1	MDS-MLD	M	78	yes	2.5	no	BCOR (21), U2AF1 (13)	normal	no
LR-MDS	1	MDS-MLD	M	90	yes	2	no	CALR (69), JAK2 (4), TET2 (42/7)	normal	no

cohort	PCA cluster	WHO classification (2016)	sex	age	anemia	IPSS-R score	trans-fusions	mutation (VAF in %)	cytogenetics	RS
**LR-MDS**	2	MDS-MLD	F	71	yes	3	yes	none	normal	no
**LR-MDS**	2	MDS-MLD	M	50	yes	2.5	no	none	normal	no
**LR-MDS**	2	MDS-MLD	M	62	yes	3.5	no	IDH1 (33), TET2 (52)	+8	no
**LR-MDS**	2	MDS-MLD	F	78	no	2.5	no	DNMT3A (4.4)	normal	no
LR-MDS	2	MDS-MLD	M	62	yes	3.5	no	none	del(7q),+1,der(1;7)	no
LR-MDS	2	MDS-MLD	M	68	yes	2.5	no	none	normal	no
LR-MDS	2	MDS-MLD	M	59	yes	2	yes	TET2 (2.7)	normal	no
LR-MDS	2	MDS-RS-MLD	F	73	yes	2	no	RAD21 (14), SF3B1 (16)	normal	yes
LR-MDS	2	MDS-U	M	70	yes	3.5	yes	TP53 (7.8)	normal	no
LR-MDS	2	MDS-MLD	M	76	yes	2	yes	CUX1 (60), TET2 (44), U2AF1 (45), ZRSR2 (61)	normal	no
LR-MDS	2	MDS-MLD	M	76	yes	1	no	BRCC3 (22), RUNX1 (32.2), SRSF2 (34.4), TET2 (30/35.8)	normal	no
LR-MDS	2	MDS-MLD	M	62	yes	3	no	none	+mar	no
LR-MDS	2	MDS EB1	F	86	yes	3	yes	CUX1 (13), SF3B1 (45), TET2 (40)	normal	yes
LR-MDS	2	MDS EB1	M	84	yes	3.5	no	SRSF2 (39), TET2 (33.6)	normal	yes
LR-MDS	2	MDS EB1	F	73	yes	3.5	no	CUX1 (44), TP53 (10.3)	normal	no
**LR-MDS**	2	MDS del(5q)	F	54	yes	2	no	DNMT3A (6)	del(5q)	no
**LR-MDS**	2	MDS del(5q)	F	68	yes	2	no	ASXL1 (18), CUX1 (6)	del(5q)	no
**LR-MDS**	2	MDS del(5q)	F	52	no	3	no	none	del(5q)	no
LR-MDS	2	MDS del(5q)	F	85	yes	3	yes	none	del(5q),+8	no
LR-MDS	2	MDS del(5q)	F	81	yes	2.5	no	TET2 (14), TP53 (5)	del(5q),+8	no
LR-MDS	2	MDS del(5q)	F	71	yes	2	yes	SF3B1 (6), ZRSR2 (52)	del(5q)	no
LR-MDS	2	MDS del(5q)	F	72	yes	2.5	no	none	del(5q)	no
LR-MDS	2	MDS del(5q)	F	49	yes	3	yes	NA	del(5q),+8	no
LR-MDS	median age [years] = 70.3 ; ratio F/M [%] = 40.4 / 59.6									
**HR-MDS**		MDS-MLD	M	36	yes	4	yes	none	normal *[1]	no
**HR-MDS**		MDS EB1	F	89	yes	6	yes	ABL1 (49), DNMT3A (29), U2AF1 (28)	-7	no
**HR-MDS**		MDS-RS-MLD	F	74	yes	4	yes	MPL (4.6), SF3B1 (41.5), TP53 (2.2)	normal	yes

cohort	PCA cluster	WHO classification (2016)	sex	age	anemia	IPSS-R score	trans-fusions	mutation (VAF in %)	cytogenetics	RS
**HR-MDS**		MDS EB2	F	70	yes	5	no	DDX41 (25.7/51.5), SRSF2 (2.5)	normal	no
**HR-MDS**		MDS EB1	F	50	yes	4.5	yes	GNAS (48.9), SRSF2 (45.2)	normal	no
HR-MDS		MDS EB2	M	51	no	4	no	DDX41 (49.5), DNMT3A (11.8), SH2B3 (48.2)	normal	no
HR-MDS		MDS EB2	M	40	yes	4.5	no	CUX1 (41)	normal	no
HR-MDS		MDS-MLD	F	71	yes	5	no	none	+8	no
HR-MDS		MDS-RS-MLD	F	70	yes	4	yes	ASXL1 (51.8), SF3B1 (42.9), TET2 (44.6/43.4/43.5/43.3)	normal	yes
HR-MDS		MDS EB1	F	73	yes	5	yes	GNAS (43.9), SF3B1 (40.3)	t(1;3)	yes
HR-MDS		MDS EB1	F	72	yes	4	no	DNMT3A (46.8), JAK2 (2.6), SF3B1 (46.7), TET2 (6.9/6.1)	normal	yes
HR-MDS		MDS del(5q)	F	42	yes	4	no	JAK2 (7)	del(5q)	no
HR-MDS		MDS del(5q)	M	71	yes	8.5	yes	TP53 (41)	complex,del(5q)	NA
HR-MDS		MDS del(5q)	F	57	yes	4	yes	none	del(5q),+8	NA
HR-MDS	median age [years] = 61.9 ; ratio F/M [%] = 71.4 / 28.6									

LR-MDS PCA clusters according to Figure 3 are indicated. Samples marked in bold were used for sorting (Figure 5). CHIP: clonal hematopoiesis of indeterminate potential, MDS: myelodysplastic neoplasms, LR-MDS: low-risk MDS, HR-MDS: high-risk MDS, F: female, M: male, NA: not available, RS: ringsideroblasts/cut-off ≥ 15%, VAF: variant allele frequency.

*[1] 6 months later +1,der(1;7)

Supplementary Table S2. Primer sequences for qRT-PCR analysis of sorted bone marrow populations.

gene	forward primer	reverse primer
U6	5´-AACGCTTCACGAATTTGCGT-3´	5´-CTCGCTTCGGCAGCACA-3´
S100A9	5´-CGGCTTTGACAGAGTGCAAG-3´	5´-GCCCCAGCTTCACAGAGTAT-3´
NLRP3	5´-CAAGCAAGATGCGGAAGCTC-3´	5´-GTCCTCCACCAGGTAGGACT-3´
PYCARD	5´-CAAGCAAGATGCGGAAGCTC-3´	5´-GTCCTCCACCAGGTAGGACT-3´
CASP1	5´-GCCCACCACTGAAAGAGTGA-3´	5´-CTTCACTTCCTGCCCACAGA-3´
IL1B	5´-TGATGGCTTATTACAGTGGCA-3´	5´-GGTGGTCGGAGATTCGTAGC-3´
IL18	5´-TGCAGTCTACACAGCTTCGG-3´	5´-ACTGGTTCAGCAGCCATCTT-3´
NLRC4	5´-GGGATCACCTTTGACCTTTCCA-3´	5´-GGGCTCGGCTATTGTCCTTT-3´

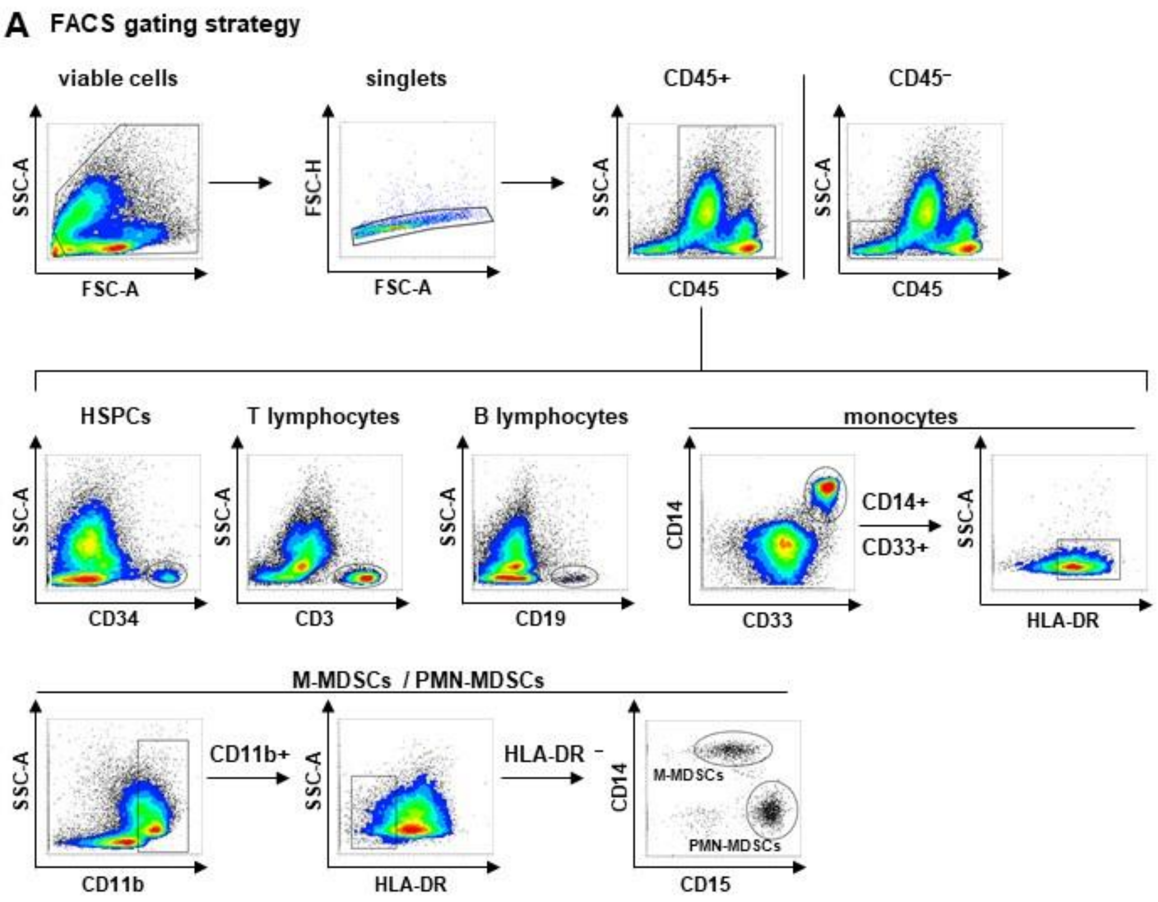

B sorted populations – marker expression and antibody specifications

population	marker expression	company #cat.nr.	fluorochrome	clone
**CD45-**	CD45-	BD #564105	PerCP-Cy5.5	HI30
**HSPCs**	CD45+	BD #564105	PerCP-Cy5.5	HI30
	CD34+	BD #345804	APC	8G12
**T lymphocytes**	CD45+	BD #564105	PerCP-Cy5.5	HI30
	CD3+	BD #345763	FITC	SK7
**B lymphocytes**	CD45+	BD #564105	PerCP-Cy5.5	HI30
	CD19+	Beckman Coulter #A07769	PE	J3-119
**monocytes**	CD45+	BD #564105	PerCP-Cy5.5	HI30
	CD14+	BD #345787	APC	MφP9
	CD33+	BD #333952	PE-Cy7	P67.6
	HLA-DR+	BD #641411	APC-H7	L243
**M-MDSCs**	CD45+	BD #564105	PerCP-Cy5.5	HI30
	CD11b+	BD #561685	PE-Cy7	ICRF44
	HLA-DR-	BD #641411	APC-H7	L243
	CD14+	BD #345784	FITC	MφP9
	CD15-/low	BD #561716	APC	HI98
**PMN-MDSCs**	CD45+	BD #564105	PerCP-Cy5.5	HI30
	CD11b+	BD #561685	PE-Cy7	ICRF44
	HLA-DR-	BD #641411	APC-H7	L243
	CD14-	BD #345784	FITC	MφP9
	CD15+	BD #561716	APC	HI98

Supplementary Figure S1. Detailed FACS gating strategy of sorted bone marrow populations.
(**A**) Detailed gating strategy of sorted bone marrow population and (**B**) corresponding marker expression per population including antibody specifications.

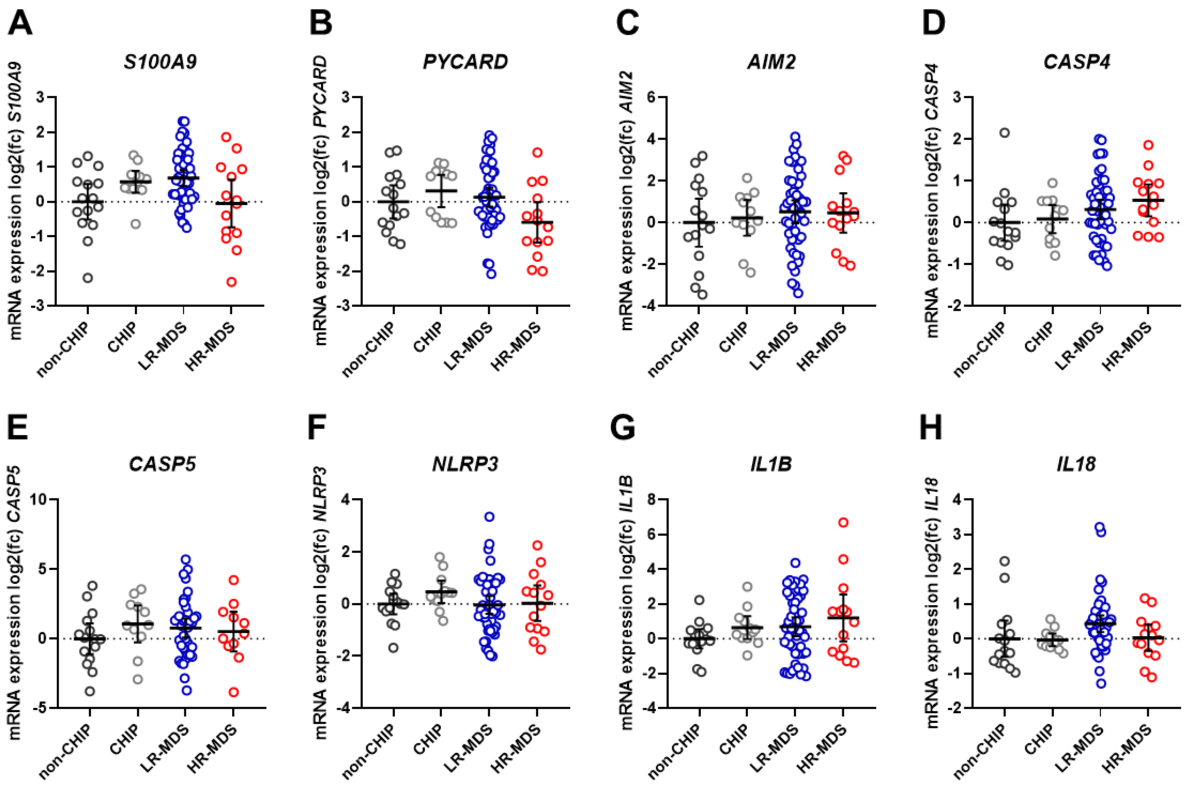

Supplementary Figure S2. Inflammasome transcript profiling in healthy individuals and MDS patients. mRNA expression values of (**A**) *S100A9*, (**B**) *PYCARD*, (**C**) *AIM2*, (**D**) *CASP4*, (**E**) *CASP5*, (**F**) *NLRP3*, (**G**) *IL1B* and (**H**) *IL18* are plotted as log2 fold changes (mean non-CHIP = 0). Horizontal and vertical bars depict the mean and 95% confidence interval, respectively. Cohorts: non-CHIP (*n* = 15), CHIP (*n* = 12), LR-MDS (*n* = 47) and HR-MDS (*n* = 14). Kruskal-Wallis test followed by Dunn's test for multiple comparisons was applied to compare differences between all groups. CHIP: clonal hematopoiesis of indeterminate potential, MDS: myelodysplastic neoplasms, LR-MDS: low-risk MDS, HR-MDS: high-risk MDS.

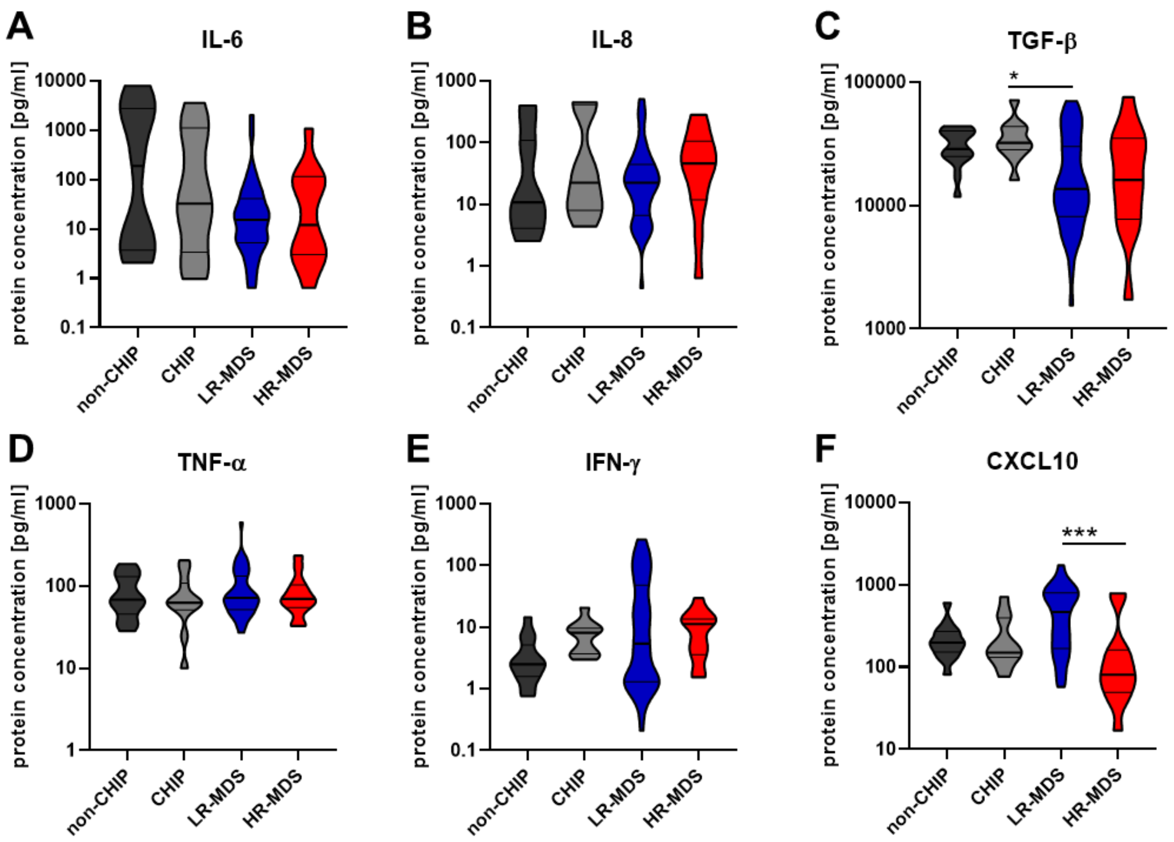

Supplementary Figure S3. Protein measurement in bone marrow plasma samples.
Violin plots of (**A**) IL-6, (**B**) IL-8, (**C**) TGF-β, (**D**) TNF-α, (**E**) IFN-γ and (**F**) CXCL10 protein concentrations in bone marrow plasma samples. Bars depict the median (bold) and quartiles. IL-10 and IL-17A were not consistently detectable (data not shown). Cohorts: non-CHIP (*n* = 15), CHIP (*n* = 12), LR-MDS (*n* = 47) and HR-MDS (*n* = 14). Kruskal-Wallis test followed by Dunn's test for multiple comparisons was applied to compare differences between all groups: * $p \leq 0.05$, ** $p \leq 0.01$, *** $p \leq 0.001$, **** $p \leq 0.0001$. CHIP: clonal hematopoiesis of indeterminate potential, MDS: myelodysplastic neoplasms, LR-MDS: low-risk MDS, HR-MDS: high-risk MDS, IL: interleukin, TGF-β: transforming growth factor-β, TNF-α: tumor necrosis factor-α, IFN-γ: interferon-γ, CXCL10: C-X-C motif chemokine ligand 10.

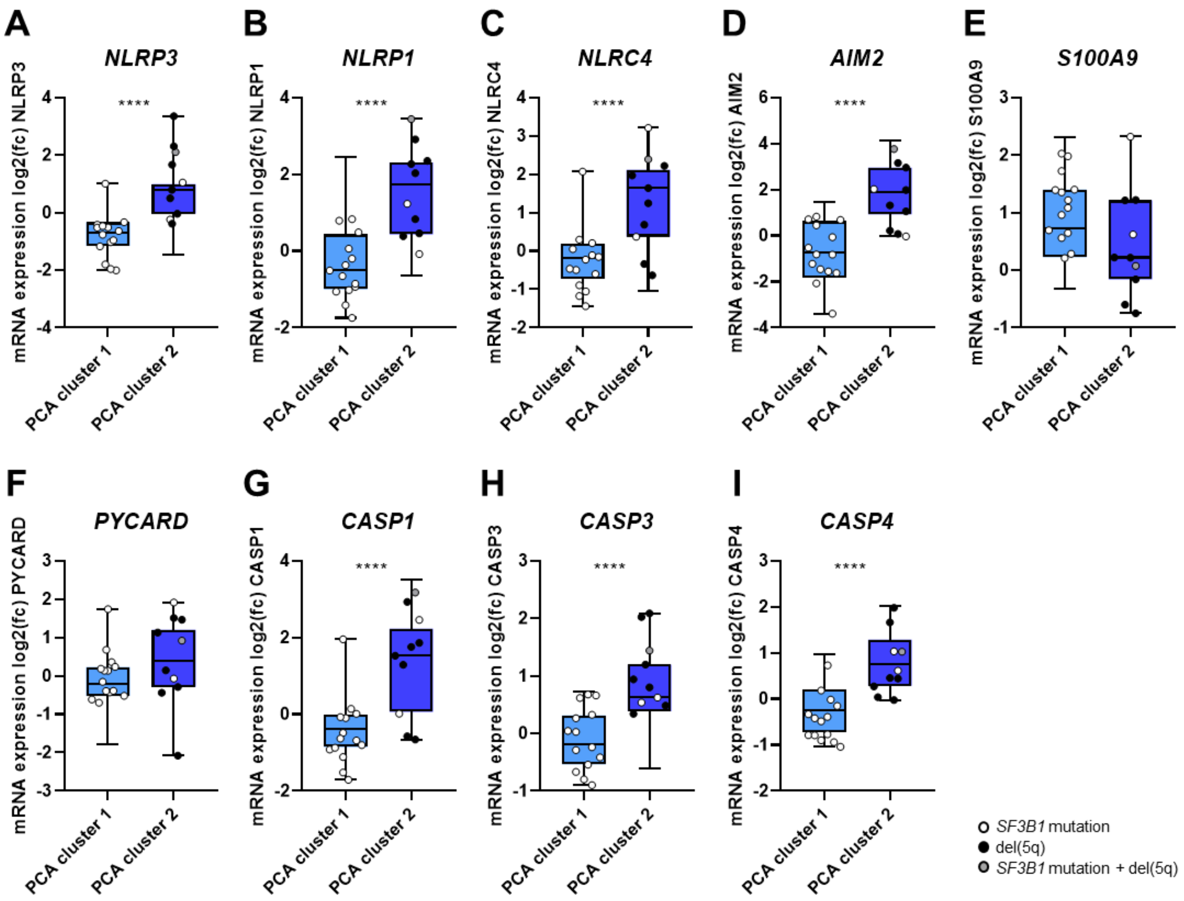

Supplementary Figure S4. Inflammasome-related gene expression per LR-MDS PCA cluster.
(**A**) *NLRP3*, (**B**) *NLRP1*, (**C**) *NLRC4*, (**D**) *AIM2*, (**E**) *S100A9*, (**F**) *PYCARD*, (**G**) *CASP1*, (**H**) *CASP3* and (**I**) *CASP4* mRNA expression values are plotted as log2 fold changes (mean non-CHIP = 0) per LR-MDS (n = 47) PCA cluster: PCA cluster 1 (n = 24), PCA cluster 2 (n = 23). Boxes show the median and whiskers show min. and max. values. Mann-Whitney test was applied to compare the difference between the PCA clusters: **** $p \leq 0.0001$.

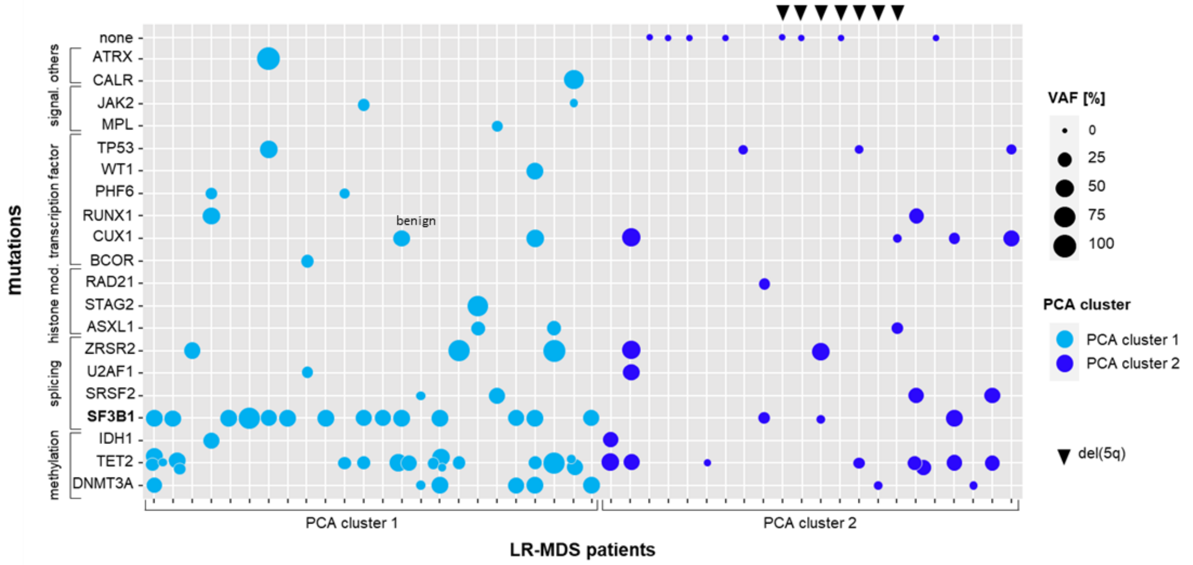

Supplementary Figure S5. Individual mutational profile of LR-MDS patients per PCA cluster. Mutations are sorted in functional groups. Del(5q) cases are marked with an arrowhead above the diagram. MDS: myelodysplastic neoplasms, LR-MDS: low-risk MDS, VAF: variant allele frequency.

gene expression in sorted populations of LR-MDS

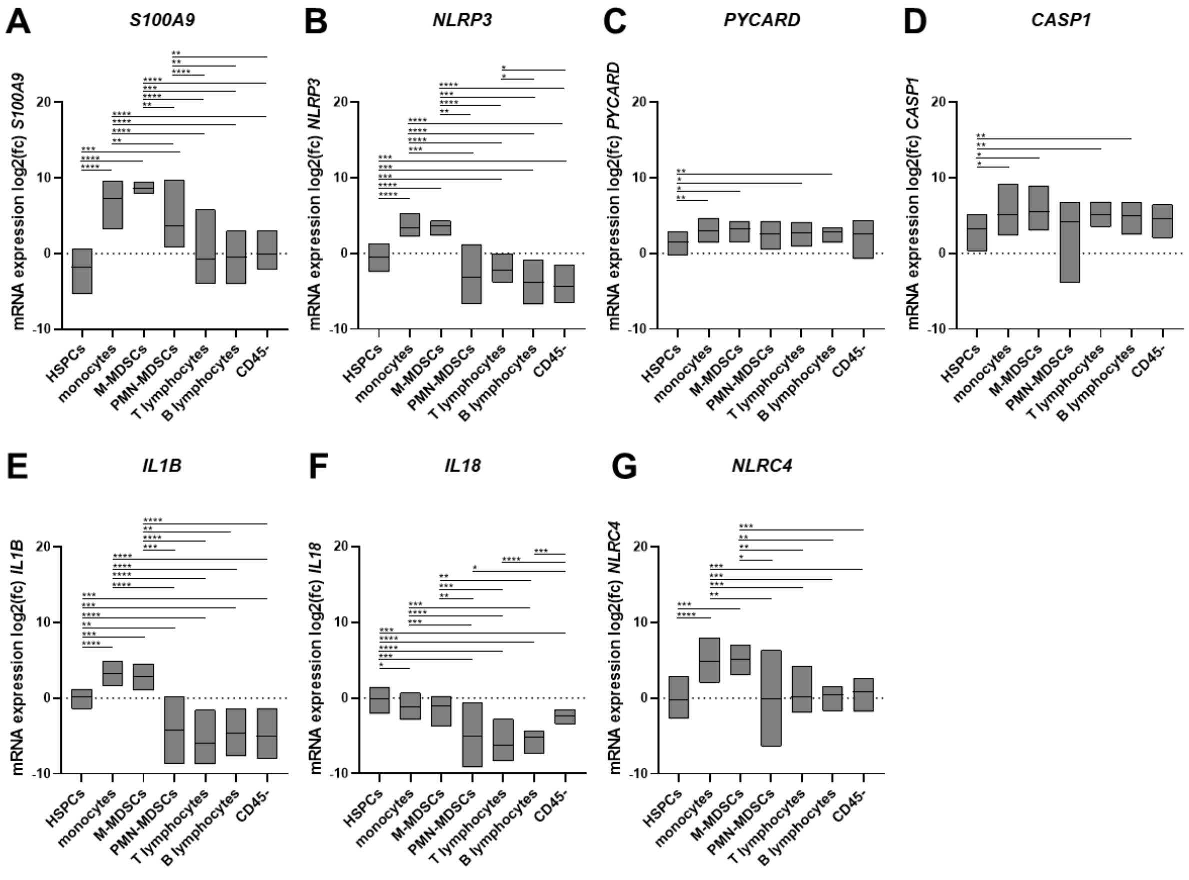

Supplementary Figure S6. Inflammasome transcript profiling in sorted bone marrow populations. (**A-G**) mRNA expression values of inflammasome-related genes (**A**) *S100A9*, (**B**) *NLRP3*, (**C**) *PYCARD*, (**D**) *CASP1*, (**E**) *IL1B*, (**F**) *IL18* and (**G**) *NLRC4* in LR-MDS patients (n = 14) are plotted as log2 fold changes (mean non-CHIP HSPCs = 0). Floating bars show min. to max. values and line shows the mean. Mixed-effects analysis with Geisser-Greenhouse correction and Tukey's multiple comparisons test were applied to compare differences between the sorted populations in LR-MDS patients: * $p \leq 0.05$, ** $p \leq 0.01$, *** $p \leq 0.001$, **** $p \leq 0.0001$.

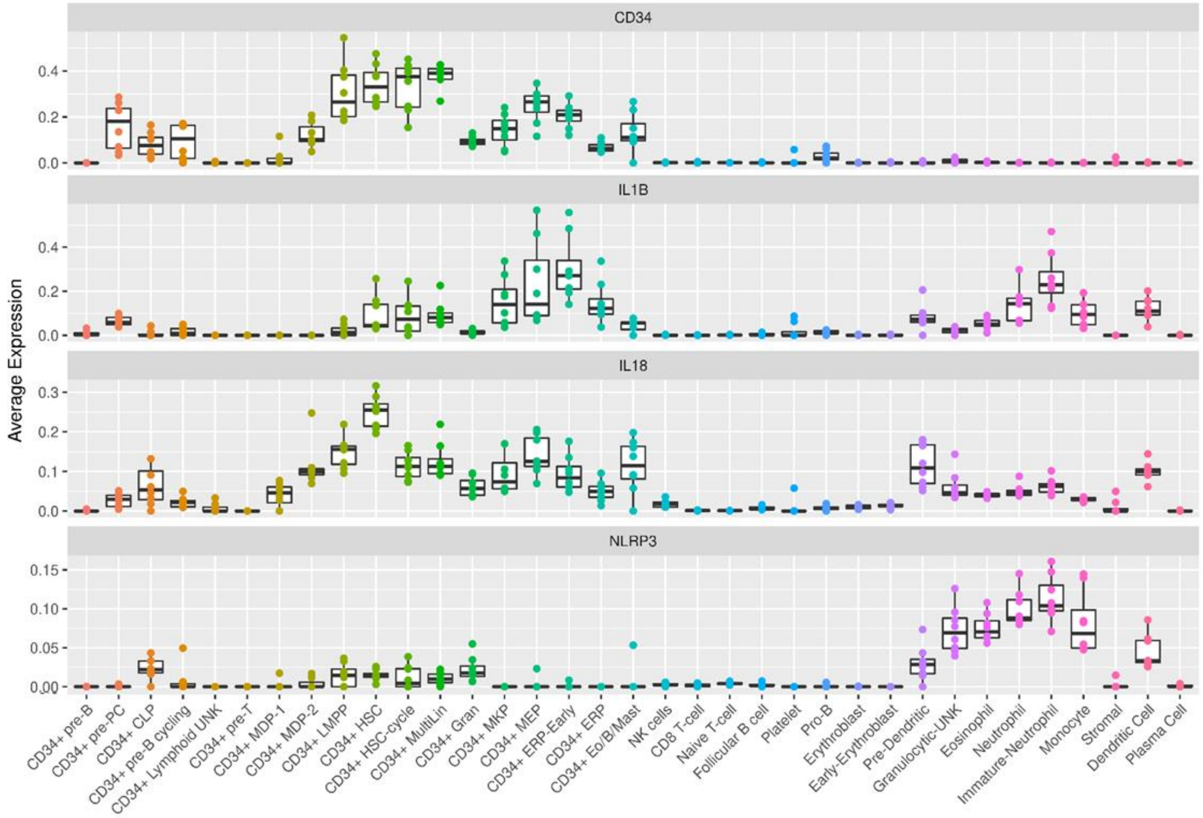

Supplementary Figure S7. Single-cell RNA-seq data of bone marrow samples from healthy donors. Single-cell RNA-seq raw data of bone marrow samples from healthy donors (n = 8) was downloaded from Human Cell Atlas[29]. The data was normalized and transformed using SCTransform*[1] by Seurat followed by cell type annotation*[2]. Average gene expression of *CD34*, *IL1B*, *IL18* and *NLRP3* was calculated as mean of gene expression for cells from the same donor. Vertical floating bars indicate the range, and top and bottom horizontal line of the box refer to the 25% and 75% quantile, while horizontal line in the box refers to the median.

*[1] Hafemeister C, Satija R. Normalization and variance stabilization of single-cell RNA-seq data using regularized negative binomial regression. Genome Biol. 2019;20(1):296. *[2] Hay SB, Ferchen K, Chetal K, Grimes HL, Salomonis N. The Human Cell Atlas bone marrow single-cell interactive web portal. Exp Hematol. 2018;68:51-61.

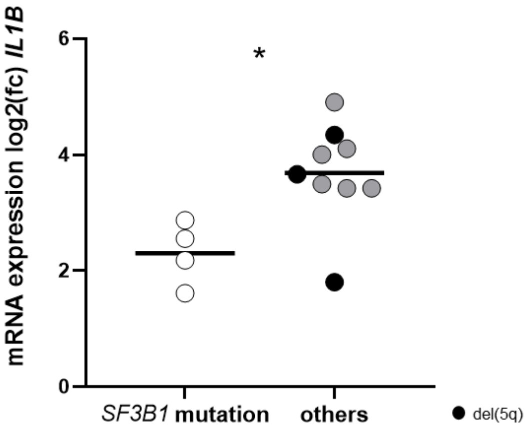

Supplementary Figure S8. *IL1B* expression in sorted bone marrow-derived monocytes.
IL1B expression in sorted monocytes of LR-MDS patients is plotted as log2 fold changes (mean non-CHIP HSPCs = 0) per MDS genotype: *SF3B1* mutation and others. Line shows the mean. Mann-Whitney test was applied to compare the difference between the MDS genotypes: * $p \leq 0.05$.

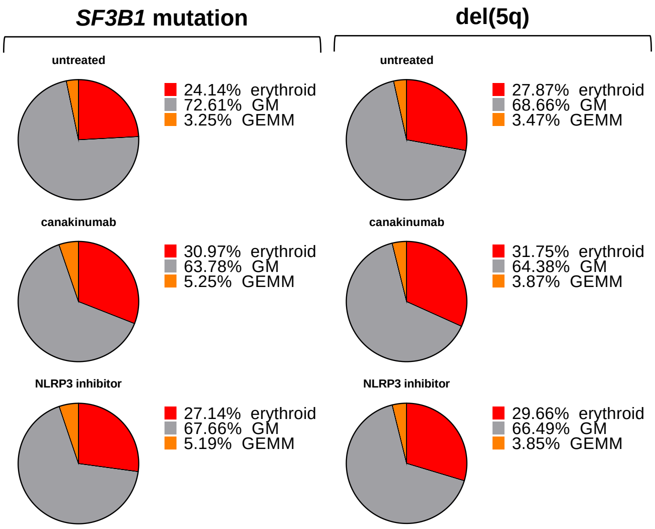

Supplementary Figure S9. Distribution of CFU colony types under *in vitro* anti-inflammatory treatment. Distribution of CFU colony types of healthy HSPCs co-cultured with monocytes from *SF3B1*-mutated (n = 3) and del(5q) (n = 3) LR-MDS patients under *in vitro* anti-inflammatory treatment with canakinumab [100 µg/ml] and the NLRP3 inhibitor IFM-2384 [10 µM] is shown in pie charts. Two-way ANOVA test followed by Tukey´s test for multiple comparisons was applied to compare differences of the colony types between the treatment conditions. GM: granulocyte–macrophage, GEMM: granulocyte–erythrocyte–macrophage–megakaryocyte.
